# Long-term outcomes of PSMA PET/CT-guided radiotherapy in biochemical failure patients post-radical prostatectomy: a 5-year follow-up analysis

**DOI:** 10.1007/s00259-025-07255-6

**Published:** 2025-04-05

**Authors:** Andrea Di Giorgio, Giambattista Siepe, Francesca Serani, Martina Di Franco, Claudio Malizia, Paolo Castellucci, Stefano Fanti, Andrea Farolfi

**Affiliations:** 1 Nuclear Medicine, Alma Mater Studiorum, University of Bologna, Via Massarenti 9, Bologna, 40138 Italy; 2 Radiation Oncology, IRCCS Azienda Ospedaliero-Universitaria di Bologna, Bologna, 40138 Italy; 3 Nuclear Medicine Unit, Department of Medicine DIMED, University-Hospital of Padova, Padova, Italy; 4 Nuclear Medicine, IRCCS, Azienda Ospedaliero-Universitaria Di Bologna, Bologna, Italy

**Keywords:** PSMA PET/CT, Salvage radiotherapy, Biochemical recurrence, Prostate Cancer

## Abstract

**Purpose:**

To evaluate the role of PSMA PET/CT-guided salvage radiotherapy (sRT) in improving long-term biochemical recurrence-free survival (bRFS) in patients with biochemical recurrence (BCR) or PSA persistence (PERS) after radical prostatectomy (RP) for localized prostate cancer.

**Methods:**

This single-center retrospective study included 100 patients with BCR or PERS after RP who underwent [⁶⁸Ga]Ga-PSMA-11 PET/CT and sRT according to EAU guidelines. The primary endpoint was bRFS (PSA ≤ 0.2 ng/ml).

**Results:**

Sixty-three patients had BCR and 37 had PERS. Fifteen patients had PSA pre-RT < 0.5 ng/ml, while 75 had PSA pre-RT ≥ 0.5 ng/ml. [⁶⁸Ga]Ga-PSMA-11 PET/CT was positive in 52 patients, with BCR patients more frequently exhibiting local recurrence while PERS patients showed more nodal involvement. Patients with PERS received sRT and androgen deprivation therapy (ADT) in 57% of cases. The hazard ratio (HR) of treatment failure for patients with PSA pre-RT ≥ 0.5 ng/ml vs. < 0.5 ng/ml was 2.2 (*p* < 0.039). With a median follow-up of 59 months, treatment failure occurred in 36% of patients, with no difference between BCR and PERS groups. Among those with treatment failure, 64% were [⁶⁸Ga]Ga-PSMA-11 PET/CT positive at recurrence, and 39% received a new PSMA PET/CT-based RT. All patients were alive at the last analysis.

**Conclusion:**

[⁶⁸Ga]Ga-PSMA-11 PET/CT-guided sRT demonstrates significant long-term efficacy in patients with BCR or PERS after RP, leading to durable PSA response and guiding further treatment decisions.

**Trial registration:**

244/2016/O/Oss8 November 2016 retrospectively registered.

## Introduction

Prostate cancer (PCa) is the second most common cancer in men worldwide, with an estimated 1.4 million new cases diagnosed in 2020 [1]. Radical prostatectomy (RP) is the standard treatment for localized PCa, with excellent long-term outcomes [2]. However, approximately 20–40% of patients will experience biochemical recurrence (BCR) after RP, defined as a rising prostate-specific antigen (PSA) level [3].

BCR after RP can be managed through various strategies, including active surveillance, salvage radiotherapy (sRT), androgen deprivation therapy (ADT), or a combination of these therapies [4]. BCR is not a uniform entity and its clinical implications are heterogeneous. Several factors influence the risk of progression and subsequent outcomes. A recent meta-analysis has identified key prognostic factors associated with distinct clinical endpoints in patients with BCR after RP or radiotherapy (RT) [4, 5]. Following RP, predictors of distant metastatic recurrence included positive surgical margins, high ISUP grade group (ISUP gg), advanced pT stage, short PSA doubling time (PSA-DT), and elevated pre-sRT PSA.

sRT is the main potentially curative option for BCR, and its efficacy has been demonstrated in several studies [6–8]. However, the optimal timing and target volume for sRT remain a matter of debate.

The role of adjuvant hormonal therapy in conjunction with sRT for BCR after primary treatment of PCa remains uncertain, with the 2024 EAU-EANM-ESTRO-ESUR-ISUP-SIOG guidelines providing a weak recommendation for its use [4].

Conventional imaging modalities, such as computed tomography (CT) and magnetic resonance imaging (MRI), have limited sensitivity in detecting recurrent PCa, especially at low PSA levels [9]. The introduction of prostate-specific membrane antigen (PSMA) PET/CT has revolutionized the management of PCa, offering superior sensitivity and specificity in detecting recurrent disease [10, 11]. PSMA PET/CT can accurately localize recurrent PCa, even at low PSA levels, allowing for more precise and targeted sRT approaches [12].

Several studies have investigated the role of PSMA PET/CT-guided sRT in improving outcomes for patients with BCR after RP [13–16]. These studies have shown promising results, with improved bRFS and overall survival (OS) compared to conventional imaging-guided sRT. However, most of these studies have relatively short follow-up periods, with the longest published follow-up to date being a median of approximately 4 years reported by Schmidt-Hegemann et al. [14].In this study, we aimed to evaluate the long-term outcomes of PSMA PET/CT-guided sRT in a cohort of patients with BCR or biochemical persistence (PERS) after RP, with a median follow-up of 5 years.

## Materials and methods

### Study population

This single-center retrospective study included 100 patients with BCR or PERS after RP who underwent [⁶⁸Ga]Ga-PSMA-11 ([^68^Ga]Ga-PSMA) PET/CT-guided sRT at our institution between January 2016 and February 2020. The inclusion criteria were RP for PCa with curative intent, BCR (PSA ≥ 0.2 ng/ml after RP) or PERS (persistent detectable PSA after RP), [⁶⁸Ga]Ga-PSMA PET/CT performed for staging before sRT, sRT based on PSMA PET/CT findings, minimum follow-up of 4 years, availability of PSA values and clinical data at follow-up. BCR was defined as a rise in PSA ≥ 0.2 ng/ml after RP, following an initial undetectable PSA. PERS was defined as a detectable PSA level ≥ 0.1 ng/ml persisting for at least 3 months after RP, despite an initial decline.

### Treatment and follow-up

All patients underwent [⁶⁸Ga]Ga-PSMA PET/CT imaging before sRT. sRT was delivered using intensity-modulated radiotherapy (IMRT) or volumetric modulated arc therapy (VMAT) techniques. SBRT was delivered using linear accelerator-based SBRT with stereotactic body frames or respiratory gating. The planning target volume (PTV) was derived from PSA value, PSA doubling time, ISUP GG, time from RP to biochemical failure and [⁶⁸Ga]Ga-PSMA PET/CT findings, including the prostate bed and/or pelvic lymph nodes. The median radiation dose was 66 Gy (IQR 64–70 Gy) delivered in 2 Gy fractions. ADT was initiated prior to radiotherapy. Following sRT, patients were followed up with regular PSA measurements and clinical examinations. bRFS was defined as PSA ≤ 0.2 ng/ml after sRT.

### PSMA PET/CT imaging

All PSMA PET/CT scans were performed using [⁶⁸Ga]Ga-PSMA. Images were acquired on a PET/CT scanner 70 min (IQR 63–79) after intravenous injection of 159 MBq (IQR 149–168,5) of [⁶⁸Ga]Ga-PSMA-11. A low-dose CT scan was performed without contrast media using 120 kV and 15–400 mA.

PET/CT images were reviewed by two experienced nuclear medicine physicians. PSMA uptake was considered positive if focal, corresponding to anatomical structures, and visually higher than background activity. miTNM classification was made according to PROMISE criteria V2 [17]. Semiquantitative analysis involved using uOmnispace software by United Imaging Healthcare for feature extraction. This software enabled automatic segmentation of areas with PSMA uptake SUVmax ≥ 2.0 in the whole body, generating volumes of interest (VOIs). A visual check by an experienced nuclear medicine physician of all VOIs followed to confirm their pathological relevance and remove any sites of physiological (kidneys, bladder, intestine, etc.) or benign uptake based on PROMISE criteria V2 [17]. To determine the total tumor burden, we calculated the PSMA total tumor volume (PSMA-TTV) by adding all PSMA-tumor values (PSMA-TV) from tumor-associated VOIs (prostatic lesion, metastatic lymph nodes, and distant metastases). To calculate the total PSMA uptake (PSMA-TL) in all prostate lesions, we first multiplied the metabolic tumor volume (PSMA-TV) of each lesion by its mean standardized uptake value (SUVmean). PSMA-TL was then summed across all lesions to obtain a cumulative measure of PSMA uptake in the prostate, termed PSMA total total lesions (PSMA-TTL).

### Statistical analysis

All analyses were performed using R (version 4.3.1), with *p* < 0.05 considered statistically significant.

Continuous variables were summarized as medians and IQR, while categorical variables were presented as frequencies and percentages. Patient characteristics were summarized using descriptive statistics, including age, PSA levels at the following time points: pre- and post-RP, pre-sRT, and at last follow-up; tumor stage, nodal involvement, surgical margins, and ISUP grade. The median follow-up time was calculated using the Kaplan-Meier method.

Comparisons between the BCR and PERS groups were performed using the Mann-Whitney U test for continuous variables and the chi-square test or Fisher’s exact test for categorical variables.

Kaplan-Meier survival curves were generated to estimate bRFS, and differences between groups were assessed using the log-rank test.

Univariate Cox proportional hazards regression models were used to identify predictors of bRFS. Variables included in the univariate analysis were age, PSA levels at various time points, tumor stage, nodal involvement, surgical margins, ISUP grade, and treatment modalities. Variables with a p-value < 0.05 in the univariate analysis were included in the multivariate model.

## Results

### Patient characteristics

Two-hundred-forty patients were screened of whom 100 met the inclusion criteria and were included in the study. 63 (63%) of them underwent [⁶⁸Ga]Ga-PSMA PET/CT imaging due to BCR, and 37 (37%) patients due to persistent disease PERS. The median age of the patients at the time of sRT was 74 years (IQR 68–77) (Table 1). There were no significant differences in age between the BCR and PERS groups. The median PSA level at RP was 7.4 ng/mL (IQR 5–12 ng/mL). Patients with PERS had a higher median PSA at RP (7.7 ng/mL, IQR 5.9–16.3 ng/mL) compared to patients with BCR (6.4 ng/mL, IQR 4.9–9.2 ng/mL). The majority of patients had T2 (42%) or T3a (24%) disease at initial diagnosis. Patients with PERS were more likely to have T3b disease (13% vs. 4%, *p* = 0.02). Most patients had no nodal involvement (N0) disease (71%) at initial diagnosis, but patients with PERS were more likely to have nodal involvement (N+) disease (10% vs. 2%, *p* = 0.003). Overall, positive surgical margins (R+) were observed in 35% of patients. The majority of patients had ISUP Grade 1–3 (57%) at initial diagnosis. Patients with PERS were more likely to have ISUP Grade 4–5 (23% vs. 19%, *p* = 0.04). The median PSA level after RP was 0.03 ng/mL (IQR 0.01–0.23 ng/mL). Patients with PERS had a significantly higher median PSA post-RP (0.3 ng/mL, IQR 0.2–0.76 ng/mL) compared to patients with BCR (0.01 ng/mL, IQR 0.01–0.2 ng/mL). The median time from RP to [⁶⁸Ga]Ga-PSMA PET/CT was 22 months (IQR 5–57 months). Patients with PERS had a significantly shorter time from RP to [⁶⁸Ga]Ga-PSMA PET/CT (3 months, IQR 2–6 months) compared to patients with BCR (23 months, IQR 45–84 months). The median PSA level at the time of PSMA PET/CT was 0.5 ng/ml (IQR 0.28–1.1 ng/ml). Forty-one patients (41%) had PSA pre-RT < 0.5 ng/ml, while 54 patients (54%) had PSA pre-RT ≥ 0.5 ng/ml. In 5 (5%) patients, the value of PSA pre-RT was unknown.

**Table 1:** Patients’ characteristics

	All Patients	PSA relapse	PSA persistance
*n*	100	63	37
Median Age *(y)*	74 (68–77)	74 (68–77)	73 (68–77)
Median PSA at RP *(ng/mL)*	7,4 (5–12)	6,4 (4,9–9,2)	7,7 (5,9-16.3)
Tumor Stage			
T2	42	30	12
T3a	24	17	7
T3b	17	4	13
T unknown	17	12	5
Nodal Stage			
N0	71	49	22
N1	12	2	10
N unknown	17	12	5
Margin of Resection			
R+	35	24	11
R unknown	37	23	14
ISUP Grade			
1 2 3	57	43	14
4 5	42	19	23
unknown	1	1	0
Median PSA post-RP *(ng/mL)*	0,03 (0,01 − 0,23)	0,01 (0,01 − 0,2)	0,3 (0,2 − 0,76)

Since the population is 100, the number and percentage coincide. Continous data are median and interquartile range

### PSMA PET/CT findings

**Fig. 1:**
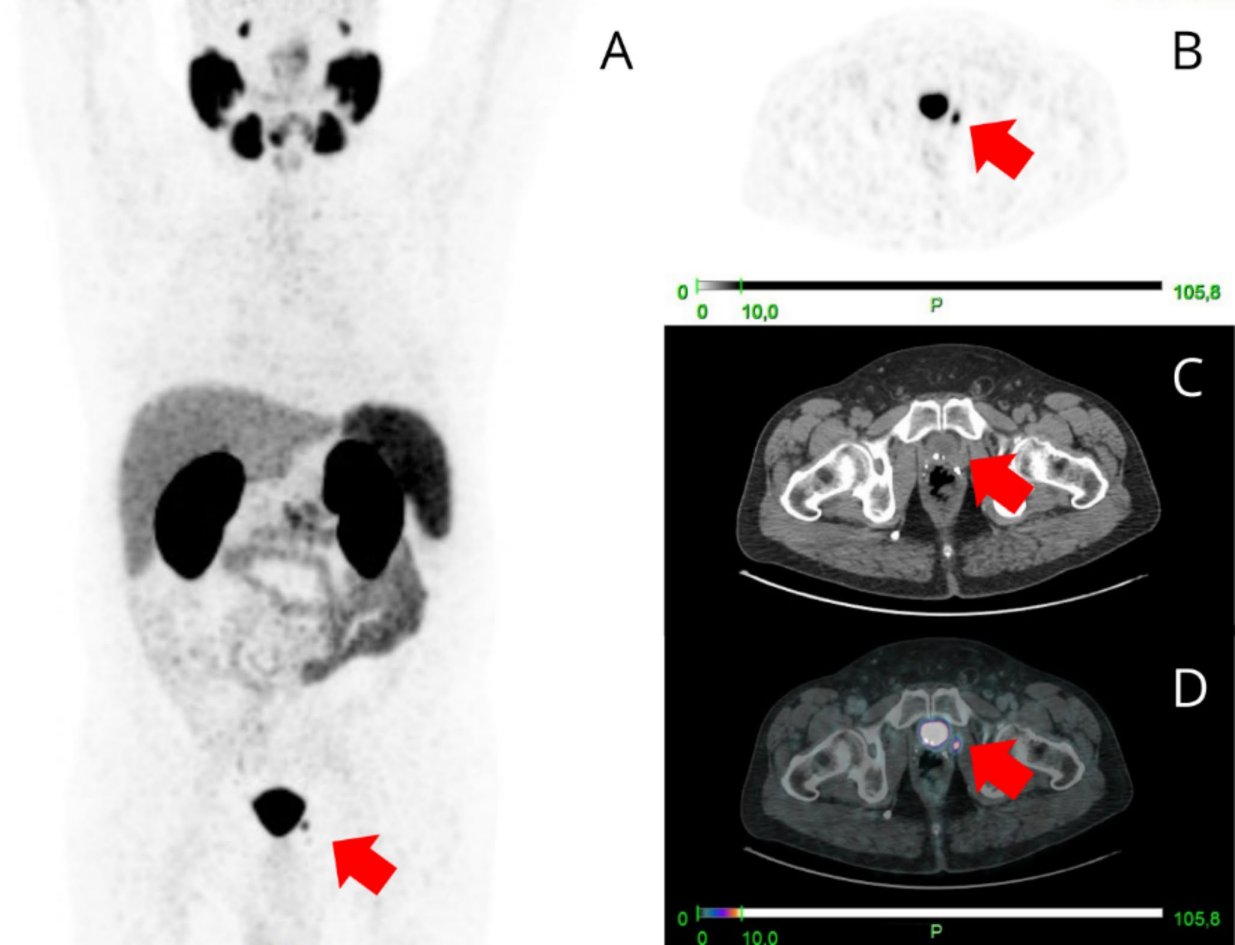
A 68-year-old male with a history of RP for PCa (initial PSA 8.4 ng/mL, Gleason score 4 + 3, ISUP gg 3, PSA post-RP 0.001 ng/mL) experienced a slow PSA rise to 1.37 ng/mL after 71 months, indicating BCR. [⁶⁸Ga]Ga-PSMA PET/CT was performed. Maximum Intensity Projection (**A**), transaxial PET image (**B**), transaxial CT image (**C**), and fused PET/CT image (**D**) revealed a macroscopic area of intense uptake (SUVmax = 11.6) in the prostate bed (red arrows). sRT was delivered to the prostate bed with concurrent short-term ADT. At 48 months post-sRT, the patient remains with an undetectable PSA

**Fig. 2:**
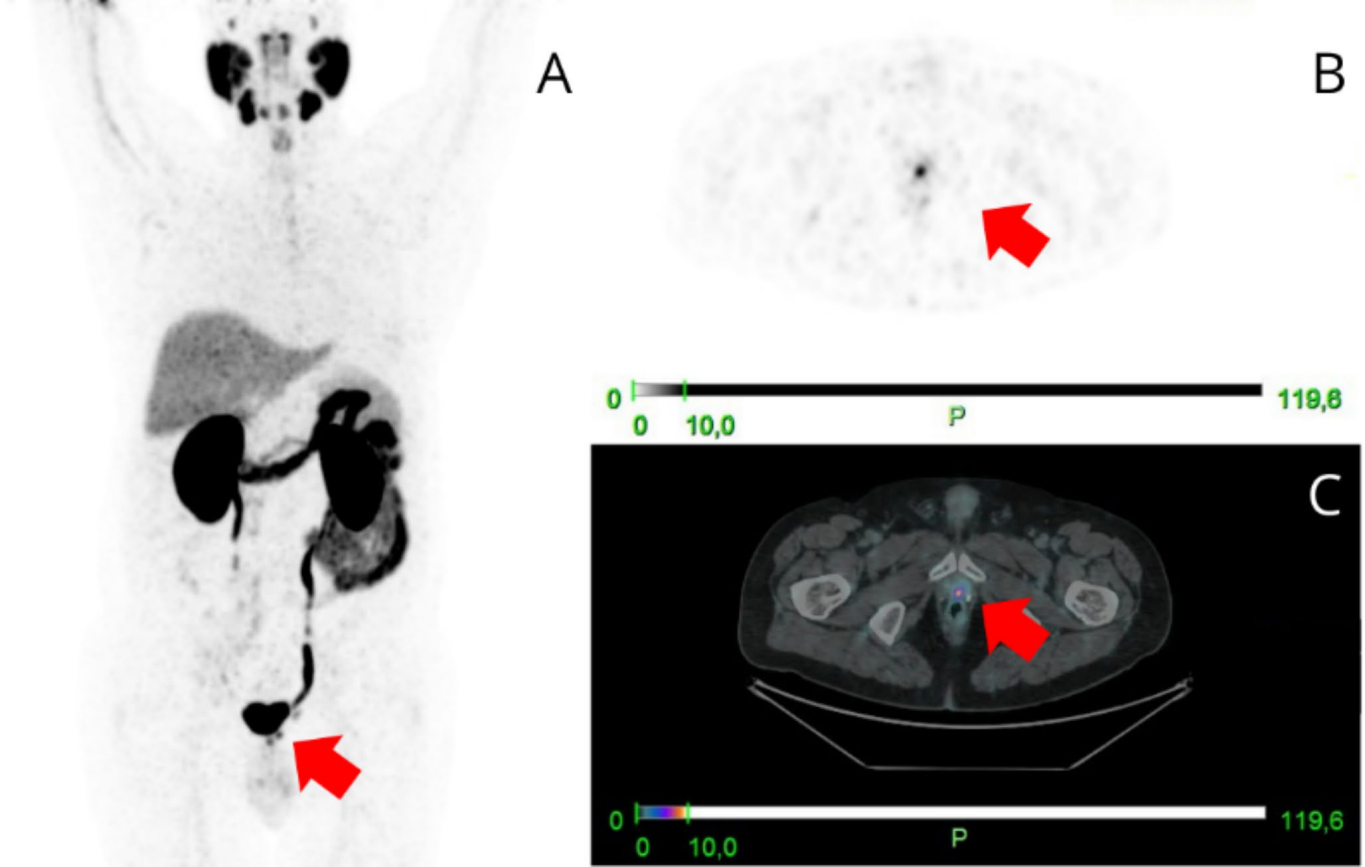
A 75-year-old male with a history of RP for PCa (iPSA 23 ng/mL, T3aR1, Gleason score 4 + 5, ISUP gg 5, PSA post-RP 0.01 ng/mL) experienced a BCR with PSA rising to 0.23 ng/mL after 5 months. [⁶⁸Ga]Ga-PSMA PET/CT revealed positive uptake in the prostate bed (SUVmax 7.1), confirming local recurrence. sRT was delivered to the prostate bed, resulting in an undetectable PSA post-RT. The patient remains with an undetectable PSA after 61 months of FU

[⁶⁸Ga]Ga-PSMA PET/CT imaging was performed at a median of 22 months (IQR 5–57) following radical prostatectomy. [⁶⁸Ga]Ga-PSMA PET/CT was positive in 52 patients (52%) (Table 2). The scan identified the site of recurrence in 28 BCR (44%) and in 24 PERS (65%). The most frequent site of recurrence in the BCR group was the prostate bed (12 in BCR vs. 3 in PERS; 43% vs. 12%), while in the PERS group, the lymph nodes were the most common site of disease (18 PERS vs. 3 BCR; 64% vs. 11%) (Figs. 1 and 2). A total of 5 (5%)patients presented with distant metastases on [⁶⁸Ga]Ga-PSMA PET/CT: 3 (3%) with nodal metastases, 2 (2%) with bone metastases, and 1 (1%) with visceral metastases. The median PSMA-TTV was 2.3 cm³ (IQR 0.9-4.2cm^3^), while the median PSMA-TTL was 7.6 cm³ (IQR 2.5-16.5cm^3^).

**Table 2:** [^68^Ga]Ga- PSMA PET/CT findings and treatment summary

	All patients (*n* = 100)		PSA relapse (*n* = 63)	PSA persistance (*n* = 37)
Median time from RP to PET/CT *(mo)*	22 (5–57)		23 (45–84)	3 (2–6)
Median PSA at PET/CT *(ng/mL)*	0,5 (0,28 − 1,1)		0,41 (0,27 − 1,1)	0,57 (0,38 − 1)
Median PSA in PET/CT + *(ng/mL)*	0,75 (0,37 − 1,34)		0,89 (0,35 − 1,23)	0,73 (0,43 − 1,75)
Median PSA in PET/CT - *(ng/mL)*	0,39 (0,26 − 0,67)		0,37 (0,25 − 0,67)	0,46 (0,36 − 0,59)
PET/CT scan results				
negative	48		35	13
T+	15		12	3
N+	27		9	18
T + and N+	1		0	1
M+	3		2	1
T + and M+	1		1	0
N + and M+	3		2	1
1st Treatment				
RT + OT	43		22	21
RT only	57		41	16
RT type				
RT to fossa only	63		44	19
SBRT only	26		14	12
RT to fossa and SBRT	11		5	6
Since the population is 100, the number and percentage coincide.				
2nd PET/CT scan	All patients		PSA relapse	PSA persistance
*n*	36*		30 (83%)	5 (17%)
Median PSA at PET/CT *(ng/mL)*	0,32 (0,29 − 0,92)		0,32 (0,29 − 0,92)	na
Months from treatment to PET/CT *(mo)*	30 (17,5–47,9)			
2nd PET /CT scan results				
negative	12 (33%)			
positive				
T+	3 (8%)			
N+	7 (19%)			
M+	12 (33%)			
N + and M+	1 (3%)			
T+, N + and M+	1 (3%)			
2nd treatment	All patients		PET/CT scan +	PET/CT scan -
*n*	34		21 (62% )	13 (38%)
no change of treatment	14 (41% )		4 (12%	10(29%)
New RT treatment (fossa and/or SBRT)	14 (41% )		13 (38% )	1 (3%)
Only medical treatment	6 (18% )		4 (12% )	2 (6%)
	Median			
Time of Follow-up *(m)*	58,5 (51–67)			
PSA at last Follow-up *(ng/mL)*	0,01 (0,01 − 0,1)			

*1 patient performed PET/CT scan for clinical evidences and no BCR (PET/CT scan resulted negative)

miTNM classification made according to PROMISE criteria V2(17). Continuous data are median and range

### Treatment outcomes

The initial treatment for BCR varied according to [⁶⁸Ga]Ga-PSMA PET/CT findings and patient characteristics. Overall, 63% of patients sRT to the prostate bed with or without androgen deprivation therapy (ADT), 11% underwent sRT combined with stereotactic body radiotherapy (SBRT), and 26% received SBRT alone (Table 3). Patients with negative [⁶⁸Ga]Ga-PSMA PET/CT findings underwent sRT to the prostate bed alone. Patients with positive [⁶⁸Ga]Ga-PSMA PET/CT findings underwent RT to the prostate bed with or without a boost to macroscopic lesions or SBRT of PET-positive lymph nodes, depending on the findings. Oligometastatic patients (defined as those with ≤ 5 lesions) underwent SBRT. Concomitant ADT was administered to 43 patients (43%), with a median duration of 7 months (IQR 2–16), followed by discontinuation in 81% (35/43) of those patients. Thirty-four patients (34% ) experienced BCR after initial treatment and underwent a second [⁶⁸Ga]Ga-PSMA PET/CT scan at a median of 30 months (IQR 17.5–47.9) after treatment. The median PSA at the second [⁶⁸Ga]Ga-PSMA PET/CT was 0.32 (IQR 0.29–0.92). Based on the findings of this second scan, 14 patients (41% ) received a new course of RT or SBRT, 14 patients (41%) continued with the previous treatment, and 6 (18% ) switched to hormone therapy or androgen receptor pathway inhibitors (ARPI).

**Table 3:** Radiotherapy and ADT treatment information

	*n*.
RT to fossa only	63
SBRT only	26
RT to fossa and SBRT	11
Radiotherapy Dosage *(Gy)*	
*RT to fossa*	
<66	7
66–70	47
>70	4
unknown	16
*SBRT*	
<50	11
50–60	20
unknown	6
Adrogen Deprivation Treatment	
Patients who received ADT	43
Median duration of ADT (mo)	7 (2–16)
Patients who discontinued ADT	35

Since the population is 100, the number and percentage coincide. Continous data are median and interquartile range

At a median follow-up of 58.5 months (IQR 51–67), the median PSA was 0.01 ng/mL (IQR 0.01–0.1). All patients were alive at the time of the final analysis, which was conducted in April 2024.

### Predictors of biochemical recurrence-free survival

**Fig. 3:**
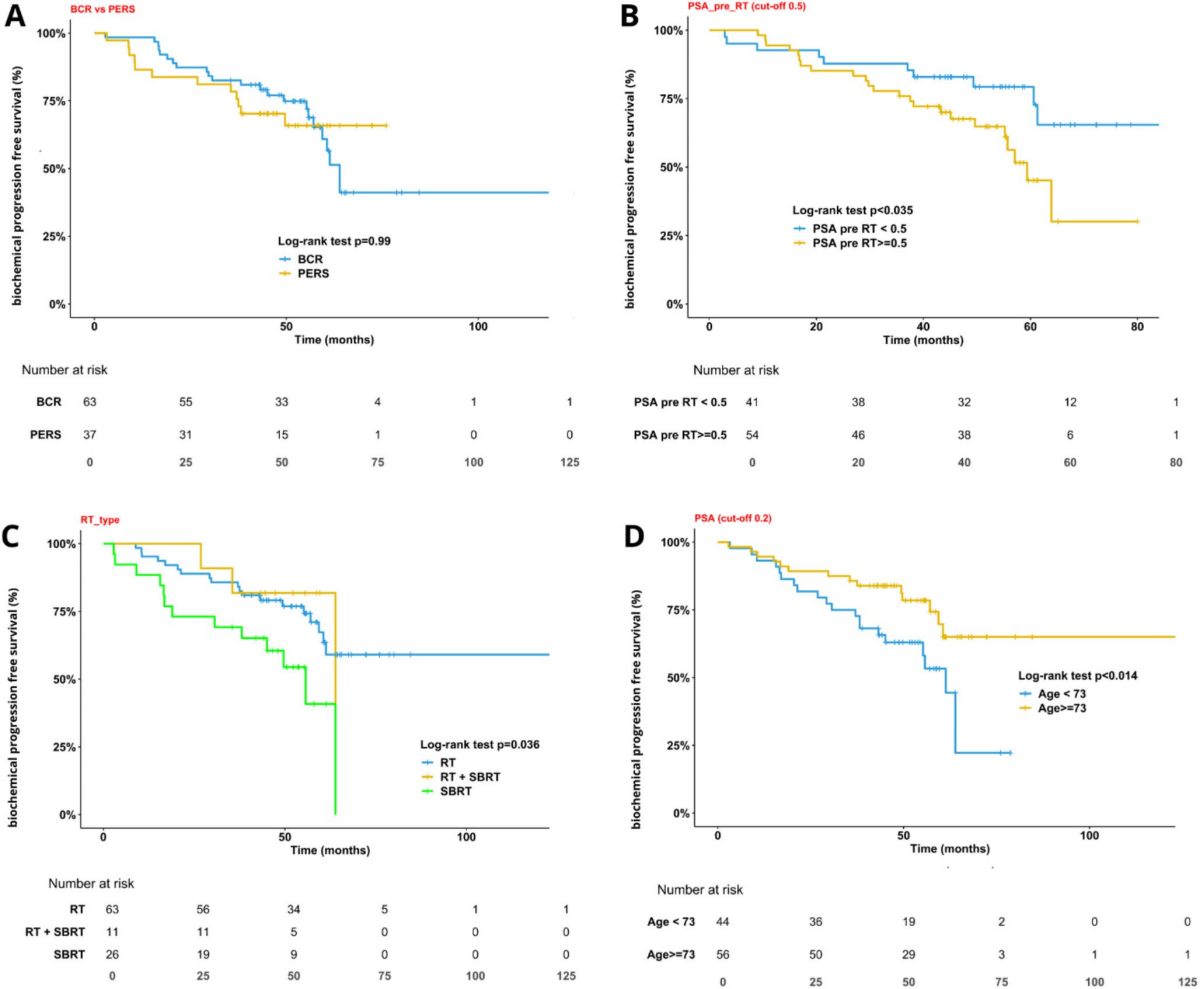
**A** bPFS (≤ 0.2 ng/ml) by biochemical recurrence status (BCR vs. PERS) at last follow-up. **B** bPFS (≤ 0.2 ng/ml) by pre-RT PSA level (< 0.5 ng/ml vs. ≥0.5 ng/ml). **C** bPFS (≤ 0.2 ng/ml) by radiotherapy type (RT to fossa vs. RT to fossa and SBRT vs. SBRT). **D** bPFS (≤ 0.2 ng/ml) by age at RT (< 73 years vs. ≥73 years)

SBRT was associated with a significantly higher risk of recurrence compared to conventional RT (*p* = 0.014, HR = 2.47,) (Fig. 3). No significant difference in recurrence rates was observed between patients with BCR and those with PERS (*p* = 0.84). On multivariate analysis, a PSA > 0.5 ng/mL at the time of salvage radiotherapy (HR = 2.2, *p* = 0.039) were independent predictors of BCR. Conversely, older age (> 73 years) was associated with a lower risk of recurrence (HR = 0.43, *p* = 0.0016). Neither PSMA-TTV nor PSMA-TTL correlated with bPFS.

## Discussion

This study evaluated the long-term outcomes of [⁶⁸Ga]Ga-PSMA PET/CT-guided sRT in a cohort of 100 patients with BCR or persistent PERS after RP. Our results demonstrate that [⁶⁸Ga]Ga-PSMA PET/CT-guided sRT is an effective treatment option for these patients, with a 5-year bRFS of 64%. These findings align with the growing body of evidence supporting the use of PSMA PET/CT to guide sRT in this patient population [13, 14]. Several studies have demonstrated improved outcomes with PSMA PET/CT-guided sRT compared to conventional imaging modalities or empirical treatment. A recent study by Zamboglou et al. further strengthens this evidence base [16]. Their analysis of a large international cohort demonstrated a significant improvement in 3-year bRFS (77% vs. 71%) and metastasis-free survival (91.2% vs. 89.2%) in patients receiving PSMA PET/CT-guided sRT compared to those receiving conventional imaging-guided sRT.

Our study builds upon previous research by providing longer-term follow-up data, with a median of 5 years. This extended observation period is crucial for assessing the durability of treatment response and identifying late recurrences. The inclusion of both BCR and PERS patients allowed for a comparison of outcomes between these two groups, which has not been extensively explored in prior studies.

Interestingly, we did not observe a significant difference in bRFS between patients with BCR and those with PERS. This suggests that [⁶⁸Ga]Ga-PSMA PET/CT-guided sRT may be an effective treatment option for both groups of patients. However, it is important to note that the PERS group in our study had a higher proportion of patients with nodal involvement on [⁶⁸Ga]Ga-PSMA PET/CT. This may explain why these patients had similar outcomes to the BCR group, despite having a lower PSA level at the time of sRT. This observation highlights the potential of [⁶⁸Ga]Ga-PSMA PET/CT to identify patients with PERS who harbor occult nodal disease and who may benefit from early sRT.

Notably, in our analysis, the use of SBRT was linked to a greater likelihood of recurrence compared to conventional radiotherapy. This association may be attributed to patients receiving SBRT having more advanced disease.

We identified several predictors of bRFS after [⁶⁸Ga]Ga-PSMA PET/CT-guided sRT. Specifically, a PSA value ≥ 0.2 ng/mL at last follow-up and a PSA > 0.5 ng/mL at the time of salvage radiotherapy were independent predictors of BCR. Conversely, older age (> 73 years) was associated with a lower risk of recurrence. These findings may help to identify patients who are at higher risk of recurrence and who may benefit from closer follow-up or more aggressive treatment.

Our findings are consistent with those reported by Schmidt-Hegemann et al. [14], who observed a 3-year bRFS rate of 73% in patients with BCR after PSMA PET/CT-guided sRT. The slightly higher bRFS rate in their study may be attributed to a shorter follow-up duration or differences in patient selection criteria. Similar to their study, we also found that a higher PSA level at the time of [⁶⁸Ga]Ga-PSMA PET/CT was associated with a worse prognosis. This highlights the importance of careful patient selection for sRT, with consideration of PSA kinetics and other risk factors. Our study further supports the use of [⁶⁸Ga]Ga-PSMA PET/CT-guided sRT in patients with PERS, as it can identify those with occult nodal disease who may benefit from early salvage treatment.

The ability of PSMA PET/CT to accurately delineate the extent of disease enables precise targeting of sRT, potentially improving local control and minimizing toxicity compared to conventional radiotherapy approaches. This precise targeting not only spares healthy tissues but also opens the door for dose escalation strategies. Incorporating additional imaging information, such as radiomic features, could further enhance the accuracy of treatment planning. For instance, Anderson et al. demonstrated the feasibility of extracting repeatable and reproducible radiomic features from MRI images of the prostate acquired using a magnetic resonance linear accelerator (RM LINAC) [18].

The concept of dose escalation using functional imaging guidance, as explored in Zapatero et al. with MRI, could be further investigated in the context of PSMA PET/CT-guided sRT [19]. Combining these approaches may allow for the delivery of higher radiation doses to PSMA-avid lesions while sparing surrounding normal tissues, potentially leading to improved treatment outcomes, particularly for patients with locally advanced or aggressive disease. This highlights the exciting potential of integrating PSMA PET/CT with advanced imaging analysis and dose escalation techniques to optimize sRT delivery and improve outcomes in prostate cancer patients.

While our study and others have shown the benefits of PSMA PET/CT-guided sRT in a retrospective setting, further prospective studies are needed to definitively establish its superiority over conventional imaging-guided approaches. Currently, the first randomized trial by Calais et al. is underway to determine if PSMA PET/CT molecular imaging can improve outcomes in patients with early BCR of PCa following RP [18, 20]. This trial aims to evaluate the success rate of sRT for recurrent PCa after prostatectomy, comparing treatment planning with and without the use of PSMA PET/CT. The results of this trial are eagerly awaited and will likely provide valuable insights into the optimal use of PSMA PET/CT in the management of recurrent prostate cancer.

It is important to note that not all studies have shown a benefit for PSMA PET/CT-guided sRT in improving bRFS. A recent multi-institutional analysis by Schmidt-Hegemann et al. found no significant improvement in bRFS with the addition of PSMA PET/CT for sRT planning in patients without evidence of nodal or distant metastases on conventional imaging [15]. This highlights the need for further research to clarify the specific patient populations that are most likely to benefit from PSMA PET/CT-guided sRT.

Our study has some limitations. First, the study is retrospective in nature, which may introduce selection bias. Second, we did not have a control group of patients who underwent conventional imaging-guided sRT. Therefore, we cannot directly compare the outcomes of [⁶⁸Ga]Ga-PSMA PET/CT-guided sRT to conventional imaging-guided sRT.Despite these limitations, our study provides valuable information on the long-term outcomes of PSMA PET/CT-guided sRT in patients with BCR or PERS after RP. Our results suggest that [⁶⁸Ga]Ga-PSMA PET/CT-guided sRT is an effective treatment option for these patients, with a high rate of bRFS at 5 years. Future prospective studies with larger cohorts and longer follow-up are needed to confirm our findings and to further evaluate the role of PSMA PET/CT-guided sRT in the management of BCR and PERS after RP.

## Conclusions

[⁶⁸Ga]Ga-PSMA PET/CT-guided sRT demonstrates significant long-term efficacy in patients experiencing BCR or PERS post-RP, eliciting a substantial PSA response over time and serving as a valuable tool in treatment management. Further research is needed to confirm our findings and to compare the outcomes of [⁶⁸Ga]Ga-PSMA PET/CT-guided sRT to conventional imaging-guided sRT.

## Data Availability

The datasets generated during and/or analysed during the current study are available from the corresponding author on reasonable request.
